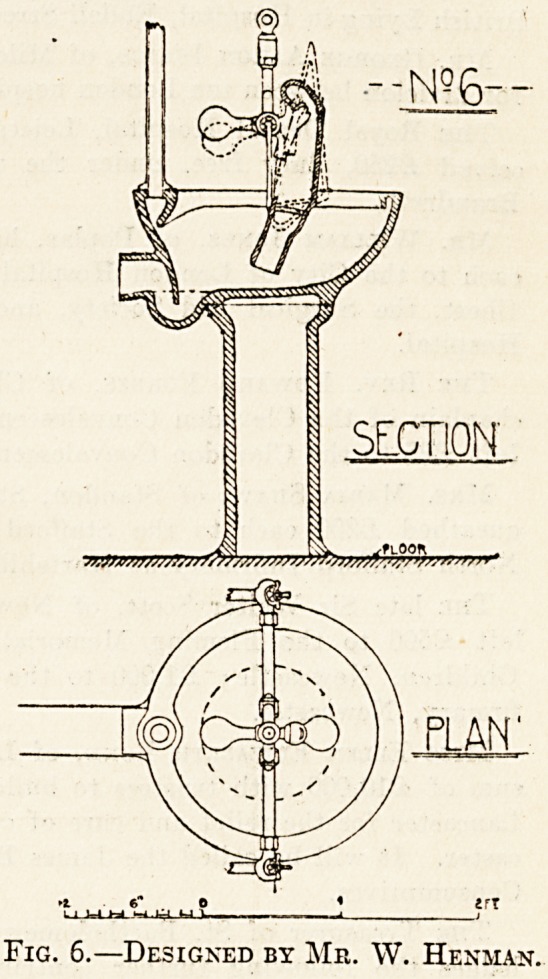# Hospital Fittings

**Published:** 1910-07-02

**Authors:** 


### HOSPITAL FITTINGS.

### BED-PAN SINKS.

The methods in use for emptying and cleansing bed-pans some 18 years ago were primitive to a degree. A common form of sink was a long hopper with a trap at the foot and a cold-water tap fixed over it. With such an appliance it is difficult to see how bed-pans were ever thoroughly cleansed, and it is clear that their use involved very serious danger to the nurse. One practical point *re* bed-pans may be mentioned. Never have hot water laid on to a slop sink, or the nurses will almost certainly suffer unduly from sore throats.

The first attempt to devise a bed-pan sink that should, while thoroughly cleansing the bed-pan, afford reasonable protection to the nurse from the danger of contamination, was a deep vessel of glazed fireclay into which the bed-pan is placed in an inverted position. (See fig. 1.) An upward jet of water is then turned on underneath the centre of the inverted bed-pan, which thoroughly scours out the vessel, whose contents are discharged through the handle. It will be seen that the position of the bed-pan in the sink is such that the outlet of the spout or handle is pointing directly to the outlet of the sink. The bed-pan is held in position by three strips of indiarubber let into grooves in the sides of the sink. A flushing rim runs all round the top of the sink and water is separately admitted to it for washing down the sides of the sink. A removable cradle is provided for urinal-bottles, and opposite the mouth of the bottle when in position on the cradle is a flushing-pipe, supplied with hot and cold water. The cradle when not in use is folded back in order to allow a bed-pan to be placed in position. The sink itself stands on a fireclay pedestal and consists, therefore, of two distinct parts, the upper part or sink proper fitting into the lower part or pedestal. Besides its primary use as a bed-pan sink it can be fitted with a hinged seat and step, and so is available for use as a w.c.

Fig. 1.—First Attempt at Bed-sink on Rational Lines.

This invention just described was, as we have said, the first attempt to construct a bed-pan sink on rational lines; and as the parent of all the numerous variations that have been devised for the same purpose, it is entitled to the credit that belongs to a pioneer in a good cause.

The defects of the apparatus are the unnecessarily large size involving an excessive area of surface for cleansing, the necessity for making it in two pieces and for carrying it down to the floor, and the cost. The means adopted for securing the bed-pan in position by rubber strips is also open to the objection that the rubber is liable to decay, and the crevices between the rubber and the glazed surface of the sink are liable to become fouled by fæcal matter.

Fig. 2.—Messrs. Dent and Hellyer's Improved Slop-sink

Fig. 2 represents a bed-pan sink modelled on the lines of the sink (fig. 1), but in which the defects noted above have been remedied. The sink is made just deep enough, but no more, to take a bed-pan and allow sufficient room for a flushing rim. The fireclay is very carefully modelled to afford a firm support for the bed-pan, and the objectionable rubber strips with their grooves are dispensed with. Thus the depth of the sink and consequently the area of surface to be cleaned is very materially reduced, and the sink becomes one piece of glazed ware held up on brackets quite free of the floor. The flush is obtained from a dual cistern, one supplying the upward flush, the other the rim-flush, each compartment of which discharges two gallons each time. The flush can either be controlled by pedals or by chain pulls, but whichever method is adopted it is important to make the distinction between the two actions as plain as possible. A convenient plan is to work the upward flush by means of a pedal, and the rim flush by means of a chain. The use of a flushing cistern is essential for two reasons: (*a*) the force of the water from a flushing cistern fixed at a suitable height above the sink, while sufficient to cleanse the bed-pan thoroughly, is not enough to affect the stability of the vessel itself, which consequently does not need to be held in position; and (*b*) if the water be laid on direct objections the force would be sufficient to lift the bed-pan out of the sink if it were not held down. One or two examples there are of sinks in which the jet of water is admitted through the handle; an arrangement which has nothing to recommend it except that of being different from anything else. Figs. 3, 4, and 5 are examples of sinks more or less adapted from Messrs. Dent and Hellyer's original type. Fig. 3 is made by Messrs. Doulton and has a second jet for cleaning urinal-bottles. The upward jet for the bed-pans is actuated by a pedal apparatus. Fig. 4 is Messrs. Jennings' pattern, and in this one the flush is applied to the nozzle of the bed-pan, so that the contents are discharged on to the surface of the sink and not directly into the trapped outgo. Fig. 5 is Messrs. Shanks' pattern and is very similar to that of Messrs. Doulton's,

Fig. 3.—Messrs. Doulton's Slop-Sink.

Fig. 4.—Messrs. Jennings' Pattern.

from the main service pipe with no intervening cistern, the whole supply might be contaminated in case of a sudden emptying of the mains. In addition to these considerations the use of an intermediate flushing cistern limited to a two-gallon flush is obligatory within the area of the Metropolitan Water Board as well as in many large towns. With the flushing-cisterns at a properly-regulated height above the sink the bed-pan can be placed in position and left, and so all unnecessary handling is avoided.

The Corbel sink, patented by Messrs. Dent and Hellyer, has been largely copied with various modifications, and in some instances that we have seen the water to the upward jet is laid on from the main service pipe. As it is quite possible that the head of water in such conditions may be anything from 20 to 60 feet it is obvious that apart from other except that the arrangement for the flushing of urinal-bottles is placed in a different part of the sink.

In fig. 6 we illustrate an apparatus designed by Mr. W. Henman, F.R.I.B.A., the architect of the Birmingham General Hospital and of the Victoria Hospital, Belfast. In designing this arrangement, which consists of two parts, the bed-pan washer and the sink, Mr. Henman's idea was to obviate the necessity for the nurse having to stand over the sink during the process of cleansing and to provide for cleansing the outside of the bed-pan as well as the inside. The arrangement is a simple one, and at the same time appears to be thoroughly effective. The bed-pan is placed on the crutch of the jet and by its own weight depresses the nozzle in such a way that the water is directed upwards into the rim and effectually scours out the contents of the pan, which are discharged into the sink and so pass away through the trap to the waste pipe. When the bed-pan is removed the nozzle is raised by the counterweight, the raised portions. This is true of the older sinks, and is due to the fact that the process of enamelling was not so well understood when the first sinks were made as it is now. With the sinks that are

Fig. 5.—Messrs. Shanks' Pattern.

Fig. 6.—Designed by Mr. W. Henman.

and the water then jets downwards and is available for cleansing the outside of the pan.

Complaint has been made that in many of the glazed ware sinks the enamel is apt to wear off on now made the enamel is proof against any damage by porcelain vessels; it is not, however, proof against the impact of a metal pail. For emptying the latter class of vessels the bed-pan sink is not, of course, intended and ought not to be used.

## Figures and Tables

**Fig. 1. f1:**
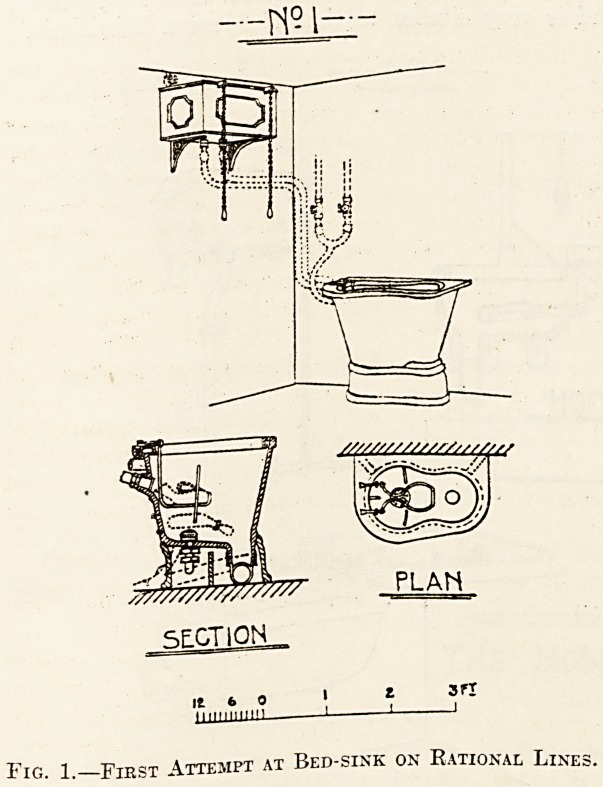


**Fig. 2. f2:**
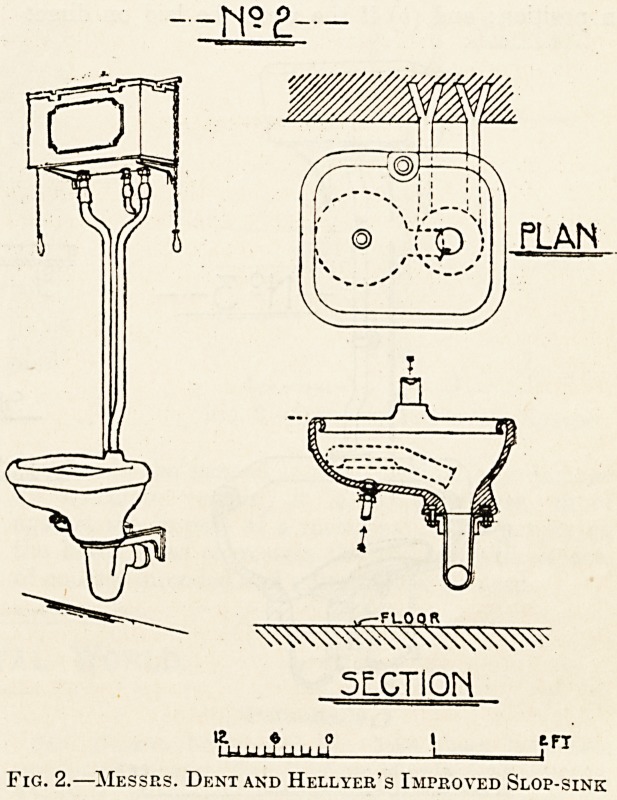


**Fig. 3. f3:**
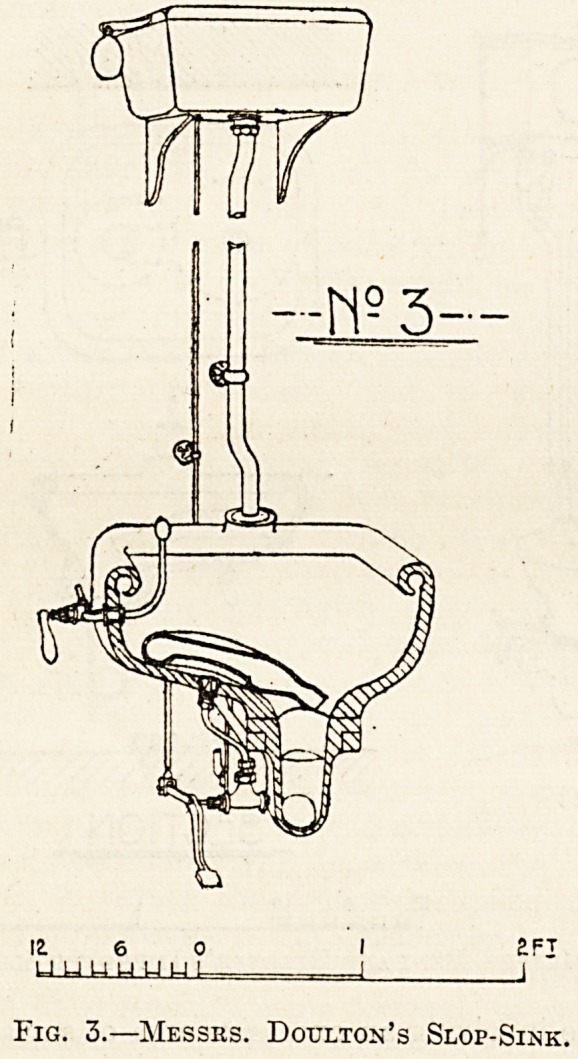


**Fig. 4. f4:**
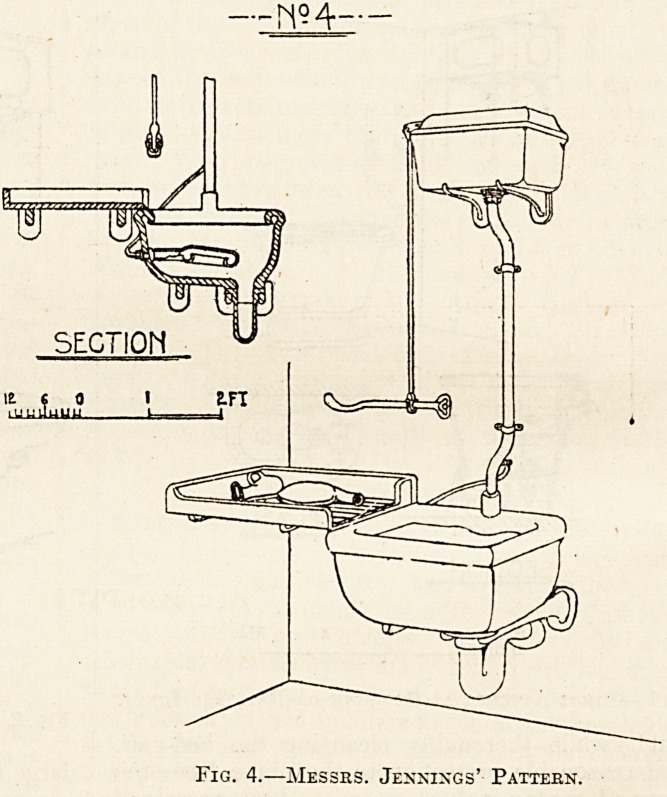


**Fig. 5. f5:**
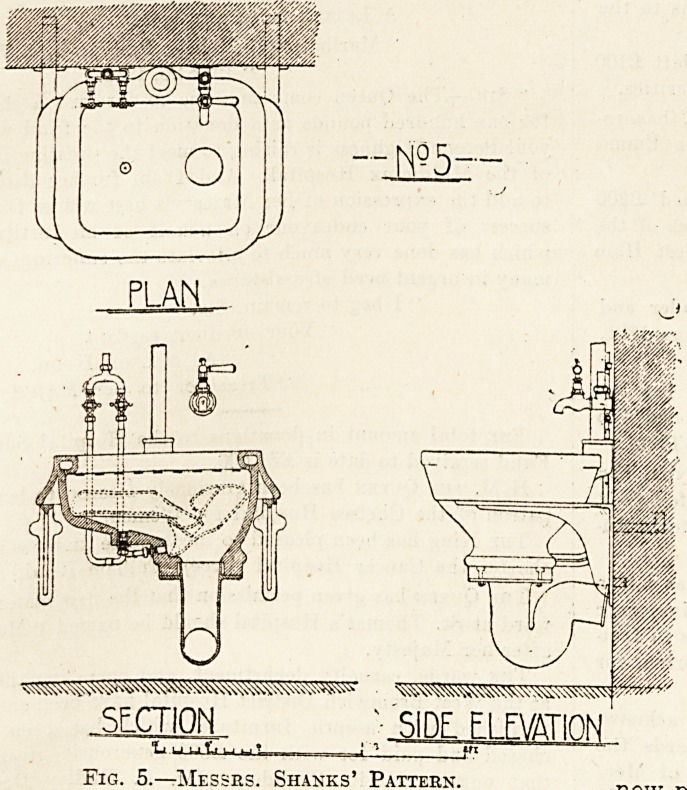


**Fig. 6. f6:**